# Structural Racism and Barriers to Breastfeeding on Chicagoland's South Side

**DOI:** 10.1089/bfm.2020.0311

**Published:** 2021-02-12

**Authors:** Margaret Butler, Jo Ann Allen, Jacqueline Hoskins-Wroten, Tytina Sanders-Bey, Ruth Nydia Venegas, Irene Webb, Kathryn Ragland

**Affiliations:** ^1^ Chicago Region Breastfeeding Task Force, Chicago, Illinois, USA.; ^2^ Department of Anthropology, Northwestern University, Evanston, Illinois, USA.; ^3^ Breastfeed Chicago, Chicago, Illinois, USA.; ^4^ Mothers' Milk Bank of the Western Great Lakes, Elk Grove, Illinois, USA.; ^5^ Department of Public Health Policy and Administration, School of Public Health, University of Illinois at Chicago, Chicago, Illinois, USA.

**Keywords:** breastfeeding, structural racism, African American women, Chicago

## Abstract

For African American (AA) families on Chicagoland's South Side who choose to breastfeed, finding and receiving services needed to reach their goals are difficult. The disparities in breastfeeding support across Chicagoland are symptomatic of inequitable health care access shaped by persisting structural racism. A number of community hospitals that once served AA families by providing easy access to care no longer exist. Recently, South Side obstetric unit and hospital closures have increased. Simultaneously, funding is increasingly competitive for community health organizations and federally qualified health centers. Institutions and agencies that do receive funding or adequately allocate funding to include lactation services cannot address breastfeeding barriers within socioeconomically marginalized communities. The unmet funding needs not only affect breastfeeding families but also impede the growth of a multilevel lactation care workforce. Finally, inconsistencies persist between breastfeeding information provided by lactation providers and delivery team care received in the hospital. Despite these barriers, we believe pathways exist to improve breastfeeding rates among South Side AA communities, such as perinatal home visiting services. Stakeholders must recognize the longstanding effects of structural racism and address the inequitable distribution of perinatal care across Chicagoland. Stakeholders must also place value in and be supportive of lactation care providers and the families they serve through both funding and policies. These changes, in addition to community-level collaboration, can improve breastfeeding rates for AA families on Chicagoland's South Side.

## Introduction

Human milk is the optimal infant feeding source due to its health benefits for both infants and the breastfeeding* parent.^1^ Exclusive breastfeeding is recommended for the first 6 months after birth, followed by continued breastfeeding for 1 year or longer, as mutually desired by the infant-parent dyad.^2^ Despite continued public health efforts to increase rates of breastfeeding, barriers persist that result in inequitable access to breastfeeding services, disproportionately affecting African American (AA) breastfeeding families and support systems.^3^

## Setting and Population

Health care access in the United States has been continuously shaped by structural racism, which encompasses the ways in which society upholds racial biases through “mutually reinforcing inequitable systems.”^4^ Chicago, Illinois, is notorious for its residential segregation, which continues to entrench both geographic separation of and disinvestment in AA communities. These policies have shaped disparities in health outcomes across the life course for AA residents,^5^ the majority of whom live on the South Side of Chicagoland.^6^ Despite just 17% of live births in Illinois being non-Hispanic Black, AA women have the highest rates of severe maternal morbidity, 101.5 per 10,000 deliveries, and the highest pregnancy-associated mortality ratio, 109 deaths per 100,000 live births.^7^ Both of these rates are three times higher than white women. In Chicago specifically, the AA infant mortality rate is 10.5 per 1,000 live births, more than three times higher than white infants.^8^ As for breastfeeding rates, the state of Illinois reports 84.2% for “ever breastfed” and 42.1% for “exclusive breastfeeding at 3 months,”^9^ far exceeding rates on the South Side of Chicago. For example, in the neighborhoods of Englewood and Roseland, with majority (95% or higher) AA populations, “ever breastfed” rates are 1.4% and 6.8%, and for “exclusive breastfeeding at 3 months,” rates are 0.6% and 4.1%, respectively.^10^ While a stark comparison, these breastfeeding rates are reflective of trends found across the United States.^3^

## Hospital Closures and Funding Priorities

More than 98% of deliveries in the United States occur in hospitals,^11^ and thus breastfeeding is most often initiated in this setting. Hospital closures have occurred steadily since the 1970s across Chicagoland, primarily driven by structural changes in the health care system, rising health care costs, low Medicaid reimbursement, and bed size.^12,13^ However, socioeconomic factors of the surrounding neighborhoods, like rates of unemployment and racial composition, drive closure as well. One study found that in Chicago, neighborhoods with majority AA populations possessed smaller hospitals, which were more likely to close due to economic resources and bed size.^12^ This example clearly demonstrates the insidiousness of structural racism, with selective health care investment setting up communities for increased vulnerability when closures inevitably occur.

Perinatal health care access on Chicagoland's South Side has historically been shaped by hospital closures, notable ones include Englewood, Osteopathic, and Hyde Park hospitals. At the beginning of 2019, Chicago had 19 hospitals with obstetric (OB) units, with 6 located on the South Side (Table 1). In 2019, two South Side hospitals closed their OB units,^14^ and in 2020, two more South Side hospitals temporarily closed their OB units due to the COVID-19 pandemic,^15^ one of which will now close permanently due to low delivery rates and budgetary concerns. Finally, a third South Side hospital announced it will close permanently in 2021. These closures leave just three OB units on the South Side, compared to the six each on the North and West sides of Chicago.

**Table 1.** Hospitals with Obstetric Units in Chicago, Grouped by Region and Includes the South Suburb Hospitals Due to Their Frequent Use by South Side Residents

Region	Hospital name	Open	Details
North Side	1. Swedish Covenant Hospital		
	2. Advocate Illinois Masonic Medical Center		
	3. Amita Saint Joseph Hospital Chicago		
	4. Amita Health Resurrection Medical Center Chicago		
	5. Amita Saints Mary and Elizabeth Medical Center Chicago		
	6. Northwestern Prentice Women's Hospital		
West Side	7. Rush University Medical Center		
	8. University of Illinois Hospital		
	9. John H. Stroger Jr. Hospital of Cook County		
	10. Mount Sinai Hospital		
	11. Norwegian-American Hospital		
	12. St. Anthony Hospital		
	13. Holy Cross Hospital	Not open	Closed in 2019
South Side	14. University of Chicago Medicine		
	15. Mercy Hospital	Not open	Closing in 2021
	16. St. Bernard Hospital	Not open	Temporarily closed OB due to COVID-19 in spring 2020; October 2020: notice of permanent OB closure
	17. Jackson Park Hospital & Medical Center	Not open	Closed in 2019
	18. Roseland Community Hospital		OB concerns
	19. Advocate Trinity Hospital		Temporarily closed OB due to COVID-19 in spring 2020
South Suburbs	20. Little Company of Mary Hospital		Evergreen Park, IL
	21. Advocate Christ Medical Center		Oak Lawn, IL
	22. Ingalls Memorial Hospital		Harvey, IL
	23. Advocate South Suburban Hospital		Hazel Crest, IL
	24. MetroSouth Medical Center	Not open	Blue Island, IL; Closed 2019

OB, obstetric unit.

Simultaneously, funding has become increasingly competitive for community health organizations and federally qualified health centers, which provide health care for underserved communities on the South Side.^16^ In addition, safety-net hospitals often do not offer all possible resources and services due to their budgets being much smaller compared to larger, private, nonprofit institutions, such as university-affiliated hospitals.^17^ Institutions and agencies that do not receive funding or adequately allocate funding to include lactation services cannot properly address breastfeeding barriers within socioeconomically marginalized communities. Thus, childbearing families may not receive appropriate levels of care based on their clinical needs and perinatal care may be fragmented as birthing families seek to establish a medical home within a health care landscape rife with service challenges. Although a *maternity care desert* is defined as a county with limited access to maternity care (e.g., no hospital offering OB care, no birth center, and no OB provider),^18^ we believe this concept applies to the South Side context, where maternity care options are rapidly decreasing and families are forced to travel farther from their communities to access perinatal care and deliver their babies.

## Lactation Services Undervalued

These unmet funding needs not only affect breastfeeding families but also are likely to impede the growth of a multilevel lactation care workforce. The distribution of existing lactation providers is an important barrier in Chicagoland. As lactation providers in this context, we are concerned by the South Side's lack of International Board Certified Lactation Consultants (IBCLCs) and other lactation service providers, as well as breastfeeding support groups for AA families. Instead, we find that AA families on the South Side rely predominantly on infant feeding services from The Special Supplemental Nutrition Program for Women, Infants, and Children (WIC) program. Although still best known within AA communities as a formula supplementation service, Illinois WIC programs are actively working to improve their reputation as a breastfeeding support resource.^19^ Despite improvement and best efforts, those of us who are or have been WIC providers supporting breastfeeding have found a lack of continuity and inconsistencies regarding the information and support shared with birthing parents by health care providers in hospitals and clinics, as well as the broader community. Oftentimes at the time of delivery, health care providers in hospitals interrupt or delay early breastfeeding efforts. This is due to various reasons, which include, but are not limited to, a lack of knowledge about breastfeeding best practices and the undervaluing of breastfeeding.^20,21^ In our opinion, this mismatch in the valuation of breastfeeding between lactation providers and the hospital delivery team is a key space where interventions are needed to improve lactation education for health care workers, as well as lactation support for families.

## Improving Breastfeeding Rates

Despite the hospital closures and funding challenges outlined above, we believe there are pathways through which breastfeeding rates can be improved for AA families on Chicagoland's South Side. Home visiting services for childbearing families are an intervention we want to highlight due to the gaps in perinatal care and breastfeeding support these services can fill, while simultaneously easing the burdens birthing families face when seeking out and accessing care.^22^ Six programs offer home visiting services in Chicago and most of these programs are funded by state and federal government: Chicago Family Case Management, Early Intervention, Family Focus, Healthy Start, Healthy Families Illinois, and Family Connects Chicago. Most of these programs focus on high-risk populations; therefore, we highlight the efforts of Family Connects Chicago, a universal postpartum nurse home visiting service for families of newborns, with visits occurring 2–3 weeks postpartum and is currently being piloted in the city.^23^ Benefits of a universal service include the reduction of stigma, assessing risk across the population, and ensuring those who need specialized care get connected to resources.^24^

Home visit services can aid in the creation of a broader network of care for Chicagoland families that encompasses breastfeeding support. The nurses conducting home visits for Family Connects have lactation training and can refer participants to lactation care providers and/or WIC services. It is important to note that home visit services must engage end users and key stakeholders to develop service components that actualize their needs, while also evaluating the service to maintain quality of care, and advocating for policies and funding that sustains long-term support.

## Conclusion

We believe more investment from government at federal, state, and local levels, as well as health care institutions and other key stakeholders on Chicagoland's South Side is necessary to achieve increased rates of breastfeeding among AA communities. These entities must recognize the longstanding effects of structural racism on the health of the communities they serve, advocate for more equitable distribution of hospitals with OB units and perinatal care services, as well as value and support the work of lactation services and support systems through both funding and policies. This will entail meeting the needs defined by both providers and the members of communities being served.^21^ It is our opinion that collaborative efforts between lactation care providers and social services like home visiting can transform how breastfeeding is conceived of, as well as improve breastfeeding rates for AA families on Chicagoland's South Side.

## Notes

^*^ While the term breastfeeding is used throughout this piece, we recognize that human milk feeding is practiced in various forms that differ from feeding human milk directly from the breast (e.g., chestfeeding, exclusive pumping, and donor milk feeding).

## References

[1] Chowdhury R, Sinha B, Sankar MJ, et al. Breastfeeding and maternal health outcomes: A systematic review and meta-analysis. Acta Paediatr 2015;104:96–113 26172878 10.1111/apa.13102 PMC4670483

[2] American Academy of Pediatrics. Breastfeeding and the use of human milk. Pediatrics 2012;129:e827–e841 22371471 10.1542/peds.2011-3552

[3] Louis-Jacques A, Deubel TF, Taylor M, et al. Racial and ethnic disparities in U.S. breastfeeding and implications for maternal and child health outcomes. Semin Perinatol 2017;41:299–307 28624126 10.1053/j.semperi.2017.04.007

[4] Bailey ZD, Krieger N, Agénor M, et al. Structural racism and health inequities in the USA: Evidence and interventions. Lancet 2017;389:1453–1463 28402827 10.1016/S0140-6736(17)30569-X

[5] Walek S. Large Life Expectancy Gaps in U.S. Cities Linked to Racial & Ethnic Segregation by Neighborhood. NYU Langone News. Published 2019. https://nyulangone.org/news/large-life-expectancy-gaps-us-cities-linked-racial-ethnic-segregation-neighborhood (accessed 1010, 2020.)

[6] Chicago Health Atlas. Non-Hispanic African American of Black. Chicago Health Atlas. Published 2020. https://www.chicagohealthatlas.org/indicators/non-hispanic-african-american-or-black (accessed 1010, 2020)

[7] Chicago Department of Public Health. Maternal Morbidity and Mortality in Chicago. 2019:27. https://www.chicago.gov/dam/city/depts/cdph/statistics_and_reports/CDPH-002_MaternalMortality_Databook_r4c_DIGITAL.pdf (accessed 1010, 2020.)

[8] Illinois Department of Public Health, Division of Vital Records. Chicago Infant Mortality Rate, 2010–2015. https://www.healthychicagobabies.org/wp-content/uploads/2018/08/IMR.pdf (accessed 826, 2020)

[9] Centers for Disease Control and Prevention. Breastfeeding Report Card. Illinois Department of Public Health. 2020. https://dph.illinois.gov/content/breastfeeding-data-breastfeeding-report-card-dnpao-cdc (accessed 1010, 2020)

[10] Illinois Department of Human Services. IDHS: FY2018 Annual WIC Breastfeeding Report. 2018. https://www.dhs.state.il.us/page.aspx?item=112838 (accessed 1010, 2020)

[11] MacDorman MF, Declercq E Trends and state variations in out-of-hospital births in the United States, 2004–2017. Birth 2019;46:279–288 30537156 10.1111/birt.12411 PMC6642827

[12] Almgren G, Ferguson M The urban ecology of hospital failure: hospital closures in the city of Chicago, 1970–1991. J Sociol 1999;26:5–25

[13] National Academies of Sciences Engineering and Medicine. Systemic Influences on Outcomes in Pregnancy and Childbirth. Washington, DC: National Academies Press (US), 2020

[14] Schencker L. Labor and delivery units are closing at Chicago-area hospitals Here's why. chicagotribune.com Published September 3, 2019. https://www.chicagotribune.com/business/ct-biz-obstetrics-closing-hospitals-labor-delivery-20190903-kgdgsq2ai5a7lmhmyzvago44gi-story.html (accessed 827, 2020)

[15] Glass K. When Maternity Wards in Black Neighborhoods Disappear. The New York Times. Published 5 5, 2020 https://www.nytimes.com/2020/05/05/parenting/coronavirus-black-maternal-mortality.html (accessed 1010, 2020)

[16] HRSA. Federally Qualified Health Centers. Official web site of the U.S. Health Resources & Services Administration. Published April 21, 2017. https://www.hrsa.gov/opa/eligibility-and-registration/health-centers/fqhc/index.html (accessed 103, 2020)

[17] Werner RM. Comparison of change in quality of care between safety-net and non–safety-net hospitals. JAMA 2008;299:2180 18477785 10.1001/jama.299.18.2180

[18] March of Dimes. Nowhere To Go: Maternity Care Deserts Across the U.S. 2020 Report. New York: March of Dimes, 2020.

[19] EverThrive Illinois. *Making WIC Work in Illinois: Opportunities and Recommendations for Program Improvement*. EverThrive Illinois; 2019. https://www.everthriveil.org/sites/default/files/docs/2019_MakingWICWork.pdf (accessed 1010, 2020)

[20] Yang S-F, Salamonson Y, Burns E, et al. Breastfeeding knowledge and attitudes of health professional students: A systematic review. Int Breastfeed J 2018;13:8 29483935 10.1186/s13006-018-0153-1 PMC5819656

[21] Johnson AM, Kirk R, Rooks AJ, et al. Enhancing breastfeeding through healthcare support: Results from a focus group study of African American mothers. Matern Child Health J 2016;20(Suppl 1):92–102 27449776 10.1007/s10995-016-2085-y PMC5290044

[22] McGinnis S, Lee E, Kirkland K, et al. Let's talk about breastfeeding: The importance of delivering a message in a home visiting program. Am J Health Promot 2018;32:989–996 28830205 10.1177/0890117117723802

[23] Chicago Department of Public Health. Family Connects Chicago. Published 2020. https://www.chicago.gov/content/city/en/depts/cdph/provdrs/healthymothers_and_babies/svcs/family-connects-chicago.html (accessed 1114, 2020)

[24] Handler A, Zimmerman K, Dominik B, et al. Illinois Family Connects Early Evaluation Report. University of Illinois at Chicago 2018. https://www.theounce.org/wp-content/uploads/2019/03/Family-Connects-IL-University-of-Illlinois-Formative-Evaluation.pdf (accessed 1114, 2020)

